# Supplementary material for: The Systems Biology Research Tool: evolvable open-source software
Source: BMC Syst Biol. 2008 Jun 29;2:55. doi: 10.1186/1752-0509-2-55 (PMC2446383; doi:10.1186/1752-0509-2-55)
Supplement: Additional file 1 — SBRT Archive. An archive of the current version of the Systems Biology Research Tool. [file 1752-0509-2-55-S1.zip › sbrt-1.4.0/doc/developers_guide/api/serialized-form.html]

Serialized Form


|  |  |  |
| --- | --- | --- |
| **Overview** \| Package \| Class \| Use \| **Tree** \| **Deprecated** \| **Index** \| **Help** | |  |
| PREV   NEXT | **FRAMES**    **NO FRAMES**     **All Classes** |  |


---


# Serialized Form


---

**Package** **sbrt**

**Class sbrt.FatalException extends java.lang.RuntimeException implements Serializable**

**serialVersionUID:** 1L

---

**Package** **sbrt.ext**

**Class sbrt.ext.ExternalSoftwareException extends java.lang.RuntimeException implements Serializable**

**serialVersionUID:** 1L

---

**Package** **sbrt.ext.mma**

**Class sbrt.ext.mma.MathematicaException extends java.lang.RuntimeException implements Serializable**

**serialVersionUID:** 1L

**Serialized Fields**

### cause

```
com.wolfram.jlink.MathLinkException cause
```

---

**Package** **sbrt.ext.R**

**Class sbrt.ext.R.JriException extends java.lang.RuntimeException implements Serializable**

**serialVersionUID:** 1L

---

**Package** **sbrt.kernel.fba.comp**

**Class sbrt.kernel.fba.comp.FbaOptException extends java.lang.RuntimeException implements Serializable**

**serialVersionUID:** 1L

**Serialized Fields**

### objectiveFunction

```
MathExpr<V> objectiveFunction
```

---

### sense

```
OptimizationSense sense
```

---

### status

```
ProgramSolverStatus status
```

**Class sbrt.kernel.fba.comp.FluxCapException extends java.lang.RuntimeException implements Serializable**

**serialVersionUID:** 1L

**Serialized Fields**

### fluxCap

```
MathExpr<V> fluxCap
```

---

### reactionName

```
java.lang.String reactionName
```

**Class sbrt.kernel.fba.comp.FluxRangeException extends java.lang.RuntimeException implements Serializable**

**serialVersionUID:** 1L

**Serialized Fields**

### rangeCutoff

```
double rangeCutoff
```

---

### offender

```
java.lang.Object offender
```

**Class sbrt.kernel.fba.comp.InvalidFluxVectorException extends java.lang.RuntimeException implements Serializable**

**serialVersionUID:** 1L

**Serialized Fields**

### fluxVector

```
UnmodifiableMap<K,V> fluxVector
```

---

**Package** **sbrt.kernel.io**

**Class sbrt.kernel.io.ConflictingInputException extends java.lang.RuntimeException implements Serializable**

**serialVersionUID:** 1L

**Serialized Fields**

### conflicts

```
UnmodifiableList<E> conflicts
```

---

**Package** **sbrt.kernel.math**

**Class sbrt.kernel.math.ConstraintViolationException extends java.lang.RuntimeException implements Serializable**

**serialVersionUID:** 1L

**Serialized Fields**

### variable

```
java.lang.Object variable
```

---

### bounds

```
Interval bounds
```

---

### violatingValue

```
java.lang.Number violatingValue
```

**Class sbrt.kernel.math.ExceededLimitException extends java.lang.RuntimeException implements Serializable**

**serialVersionUID:** 1L

**Class sbrt.kernel.math.IllegalBoundsException extends java.lang.RuntimeException implements Serializable**

**serialVersionUID:** 1L

**Serialized Fields**

### obj

```
java.lang.Object obj
```

---

### lower

```
java.lang.Number lower
```

---

### upper

```
java.lang.Number upper
```

**Class sbrt.kernel.math.InfeasibleSolutionException extends java.lang.RuntimeException implements Serializable**

**serialVersionUID:** 1L

**Serialized Fields**

### direction

```
java.lang.Object direction
```

**Class sbrt.kernel.math.Interval extends java.lang.Object implements Serializable**

**serialVersionUID:** 1L

**Serialized Fields**

### lowerBound

```
double lowerBound
```

---

### upperBound

```
double upperBound
```

**Class sbrt.kernel.math.InvalidPointException extends java.lang.RuntimeException implements Serializable**

**serialVersionUID:** 1L

**Serialized Fields**

### point

```
UnmodifiableMap<K,V> point
```

**Class sbrt.kernel.math.MissingVariableException extends java.lang.RuntimeException implements Serializable**

**serialVersionUID:** 1L

**Serialized Fields**

### variable

```
java.lang.Object variable
```

**Class sbrt.kernel.math.UnboundedVariableException extends java.lang.RuntimeException implements Serializable**

**serialVersionUID:** 1L

**Serialized Fields**

### variable

```
java.lang.Object variable
```

---

**Package** **sbrt.kernel.math.algebra**

**Class sbrt.kernel.math.algebra.IllegalFreeVariableException extends java.lang.RuntimeException implements Serializable**

**serialVersionUID:** 1L

**Serialized Fields**

### variable

```
java.lang.Object variable
```

---

**Package** **sbrt.kernel.math.geom**

**Class sbrt.kernel.math.geom.DimensionException extends java.lang.RuntimeException implements Serializable**

**serialVersionUID:** 1L

**Serialized Fields**

### dimension

```
int dimension
```

---

**Package** **sbrt.kernel.math.optimization**

**Class sbrt.kernel.math.optimization.ProgramSolverException extends ExternalSoftwareException implements Serializable**

**serialVersionUID:** 1L

---

**Package** **sbrt.shell**

**Class sbrt.shell.ApplicationException extends java.lang.RuntimeException implements Serializable**

**serialVersionUID:** 1L

---

**Package** **sbrt.shell.gui**

**Class sbrt.shell.gui.SbrtGui extends javax.swing.JPanel implements Serializable**

**serialVersionUID:** -7821511958093536087L

**Serialized Fields**

### textOutput

```
javax.swing.JTextArea textOutput
```

---

### workingDirButton

```
javax.swing.JButton workingDirButton
```

---

### executeButton

```
javax.swing.JButton executeButton
```

---

### fc

```
javax.swing.JFileChooser fc
```

---

### dc

```
javax.swing.JFileChooser dc
```

**Class sbrt.shell.gui.TextPaneGui extends javax.swing.JPanel implements Serializable**

**serialVersionUID:** -89245306682693526L

**Serialized Fields**

### textOutput

```
javax.swing.JTextPane textOutput
```

---

### workingDirButton

```
javax.swing.JButton workingDirButton
```

---

### executeButton

```
javax.swing.JButton executeButton
```

---

### fc

```
javax.swing.JFileChooser fc
```

---

### dc

```
javax.swing.JFileChooser dc
```

---

**Package** **sbrt.shell.io**

**Class sbrt.shell.io.EndOfFile extends java.lang.Object implements Serializable**

**serialVersionUID:** 0L

---

**Package** **sbrt.shell.text**

**Class sbrt.shell.text.FormatException extends ApplicationException implements Serializable**

**serialVersionUID:** 1L

---


|  |  |  |
| --- | --- | --- |
| **Overview** \| Package \| Class \| Use \| **Tree** \| **Deprecated** \| **Index** \| **Help** | |  |
| PREV   NEXT | **FRAMES**    **NO FRAMES**     **All Classes** |  |


---
